# Uncovering the neurophysiology of mood, motivation and behavioral symptoms in Parkinson’s disease through intracranial recordings

**DOI:** 10.1038/s41531-023-00567-0

**Published:** 2023-09-21

**Authors:** Lucia Ricciardi, Matthew Apps, Simon Little

**Affiliations:** 1 Neurosciences Research Centre, Molecular and Clinical Sciences Research Institute, St George’s University of London, London, UK; 2 Centre for Human Brain Health, School of Psychology, University of Birmingham, Birmingham, UK; 3 Movement Disorders and Neuromodulation Centre, University of California San Francisco, San Francisco, CA USA

**Keywords:** Neurology, Parkinson's disease

## Abstract

Neuropsychiatric mood and motivation symptoms (depression, anxiety, apathy, impulse control disorders) in Parkinson’s disease (PD) are highly disabling, difficult to treat and exacerbated by current medications and deep brain stimulation therapies. High-resolution intracranial recording techniques have the potential to undercover the network dysfunction and cognitive processes that drive these symptoms, towards a principled re-tuning of circuits. We highlight intracranial recording as a valuable tool for mapping and desegregating neural networks and their contribution to mood, motivation and behavioral symptoms, via the ability to dissect multiplexed overlapping spatial and temporal neural components. This technique can be powerfully combined with behavioral paradigms and emerging computational techniques to model underlying latent behavioral states. We review the literature of intracranial recording studies investigating mood, motivation and behavioral symptomatology with reference to 1) emotional processing, 2) executive control 3) subjective valuation (reward & cost evaluation) 4) motor control and 5) learning and updating. This reveals associations between different frequency specific network activities and underlying cognitive processes of reward decision making and action control. If validated, these signals represent potential computational biomarkers of motivational and behavioural states and could lead to principled therapy development for mood, motivation and behavioral symptoms in PD.

## Introduction

Parkinson’s disease (PD) is a highly disabling neurodegenerative movement disorder, affecting 1% of the population over the age of 60^1^. To date, motor symptoms of PD have been the main focus of research and therapy development. However, people with PD, which has been described as the quintessential neuropsychiatric disorder, also experience a range of disabling non-motor symptoms including mood, motivation and behaviour such as depression, anxiety, apathy and impulsive compulsive behaviours (ICB)^2^. The prevalence of mood and motivation disorders in PD is high, reaching 50% for depression and anxiety, 40% for apathy^3^ and 14–30% for ICB^2^. Yet, despite their prevalence, the complexity of these symptoms and their interaction with current treatments for motor symptoms, the circuit mechanisms underlying them remain unclear. As a result, there have to date been few successful treatment options for motivation and behavioral symptoms in PD^4,5^.

Mood and motivation disorders in PD have sometimes been broadly classified into two different extremes of a *single* spectrum with “hypodopaminergic” symptoms (depression, anxiety, and apathy) at one end and “hyperdopaminergic” symptoms (pathological impulsivity and ICB) at the other^6–8^. This simple distinction does support a significant role for dopamine in the pathophysiology of these symptoms and is based on a number of clinical and experimental observations^9–12^. However, a single dimension of behaviour is insufficient to account for the complex and multi-faceted neuropsychiatric symptoms (NPS) that occur in PD. Moreover, it is unlikely that complex mood states or behaviours map accurately onto such a simplistic schema such as a “hypo” or ‘hyper’ dopaminergic state. Rather, mood states arise from the complex interactions between systems in the brain, and likely arise from different computations that might also be reflected in distinct oscillatory processes that are present during specific behaviors. Therefore, different mood and motivational states might map onto different oscillations within the cortico-basal ganglia networks and these may go awry in PD, contributing to non-motor symptoms.

Invasive neurophysiology, and specifically intracranial recordings, provide tools for recording neural network signals that can be combined with behavioural paradigms that index distinct aspects of behaviour. Such recordings offer the potential to tap into neural synchronisation at different frequencies, and thus potentially functionally segregate processing streams that are spatially overlapping in the cortex and basal ganglia^13^.

Intracranial recordings can be performed using intraoperative microelectrode recordings of single or multiple neurons, or local field potential (LFP) recordings of aggregated neural population signals. Electrophysiological LFP activities are classically analysed with respect to partially physiological distinct power (spectral) bands including: delta (1–3 Hz), theta (4–7 Hz), alpha (8–12 Hz), beta (13–30 Hz) and gamma (35–100 Hz). Together these techniques can help identify the site, spectral signatures and precise temporal windows that are involved in specific cognitive sub-processes.

LFPs, when combined with cortical (magnetoencephalography, electroencephalographic and electrocorticography) recordings, can also disclose inter-regional (cortico-subcortical) networks expressed as rich, multiplexed, spatio-spectral components^14^. The precision of such recordings have the potential to dissociate the mechanisms underlying different symptoms, particularly when combined with experimental paradigms that can tap into the computational processes that underlie behaviour. In the future, these neurophysiology signals may serve as valuable biomarkers for principled re-tuning of unbalanced systems by targeting the particular frequency domains underlying a specific symptom. Such adaptive neurostimulation approaches are already proving highly fruitful for treating motor system impairments and here we propose a similar approach to non-motor impairments^15,16^. We focus on particular dimensions of mood, motivation and behaviour that are specifically impacted in PD and have been studied with intracranial neurophysiology. Disturbances in cognition, arousal, circadian and sleep rhythms also make a significant contribution to NPS in PD but have been covered elsewhere and so are not reviewed here^17–19^.

**Fig. 1:**
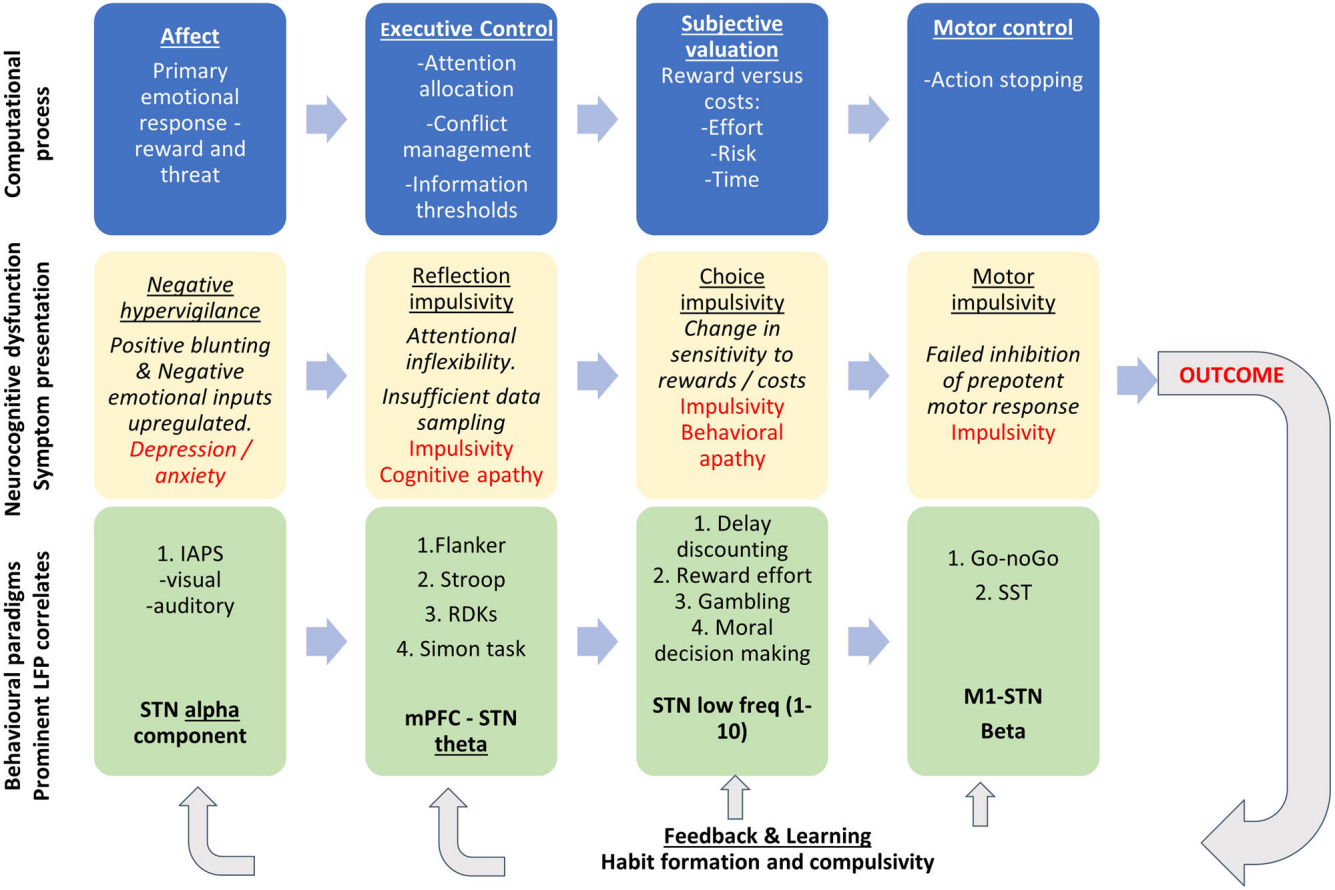
Framework for neuropsychiatric symptoms and neurophysiology in Parkinson’s disease. Schematic indicating central computational processes (top row), neurocognitive dysfunction and symptom presentation (middle row) and behavioral paradigms and frequently occurring spatio-spectral LFP correlates (bottom row). These are shown for different computational stages of processing including affect and primary emotional response, executive control of information, subjective valuation, leading to motor initiation and then motor stopping of prepotent response where required. This leads to an outcome and feedback and learning, which will update all stages of the decision cycle. All stages shown serially, although these are likely implemented in parallel.

Our framework considers symptoms and behaviour using experimental, behavioural paradigms that assay 1) emotional processing, 2) executive control 3) subjective valuation (reward and cost evaluation) 4) motor control and 5) learning and updating (Fig. 1). Here, we specifically review the mapping of these constructs onto underlying neurophysiology captured through intracranial recordings. For each cognitive process we describe neurophysiological studies that distinguish between patient groups and then focus on studies examining neurophysiological recordings using behavioral paradigms and within-subject approaches. We have included available studies using recordings from targets used in clinical practice - subthalamic nucleus (STN) is represented in a predominance of studies but we also include globus pallidus interna (GPi), ventral thalamus and pedunculopontine nucleus (PPN) in patients with PD undergoing Deep Brain Stimulation (DBS). We included both micro and macro-electrode recordings either during surgery, in the few days immediately following or in chronically implanted patients using new sensing-enabled DBS pacemakers^20^. Taken together this work suggests there is significant promise in the potential for neurophysiological recordings in patients to identify localised oscillatory signals that distinguish between different mood and motivation impairments.

### Affect and emotional processing

#### Emotional processing and relationship with depression and anxiety – across subject studies

In healthy and clinical populations it has been demonstrated that deficits in emotional processing are involved in the generation of depression and anxiety^21,22^. It is now well established that parallel segregated networks subserving limbic, associative and motor functions exist between prefrontal cortex and basal ganglia and that these are partially spatially and functionally dissociable at the level of the STN and basal ganglia^23,24^.

Single unit micro-electrode recordings have demonstrated that STN (particularly ventral STN) is associated with increased theta-alpha activity (Table 1)^25^. Further, increased resting state alpha power oscillation in the left ventral STN positively correlates with depressive symptoms in PD, whereas theta correlates negatively with both depression and anxiety^26^. LFP studies have built on this and compared intracranial neurophysiological (STN) recordings with symptoms of depression, measured with the Beck’s Depression Inventory (BDI-II)^27,28^. Overall, these studies showed reduced STN alpha reactivity (event related desynchronization) to positively valenced stimuli, and increased alpha desynchronization to negative stimuli in depressed patients with PD, effects that continue up to 3 months post-operatively^28^. STN alpha reactivity to positive emotional stimuli was also found to be enhanced (and response to negative stimuli reduced) by administration of exogenous dopamine, a relationship that was most pronounced in non-depressed patients^27^. These studies suggest that a component of the alpha rhythm in the STN may track emotional valence and that this indexes a negative emotional bias in patients with PD and depression and is modulated by dopamine.

**Table 1:** Affect and emotional processing.

Reference	Cohort	Target and medication	Task	Key findings
***Depression***				
*LFP*				
(Huebl et al. 2011)^28^	12 PD (7 with mild-moderate depression as per BDI > 9)	STN, ON med	IAPS	Significant alpha-ERD for pleasant and unpleasant trials but not for neutral ones. Smaller alpha-ERD after pleasant stimuli in PD with mild-moderate depression than PD without depression. Significant negative correlation between the alpha-ERD in unpleasant trials and BDI scores at 3 months post-DBS.
(Huebl et al. 2014)^27^	28 PD (5 developed affective disturbances post DBS): 15 ON med and 13 OFF med	STN, ON and OFF med	IAPS	Stronger decrease of alpha for pleasant stimuli in ON med condition. OFF med - larger decrease of alpha for unpleasant stimuli. Early increase of gamma in ON med group compared to OFF med group. Larger gamma for unpleasant than pleasant and neutral stimuli. Positive correlation between gamma increase and arousal values.
(Mandali et al. 2020)^37^	24 PD	STN	IAPS	Greater alpha ERD for negative and positive pictures than neutral ones in the R STN (without stimulation condition); 10 Hz stimulation increases the rating of valence for negative pictures as compared to no stimulation condition. There was a 3 way interaction between valence-arousal, frequency and BDI.
(Sun et al. 2021)^26^	21 PD (11 with depression, 10 without)	STN, OFF med	Resting state	PD with depression: increased alpha and reduced theta in the left ventral STN. Ham-D positively correlated with alpha and inversely correlated with theta.
***Emotional processing—dimensional approach***				
*Single units*				
(Sieger et al. 2015)^29^	13 PD	STN, OFF med	IAPS	17% STN neurons responded to emotional stimuli. Alpha correlated with valence or arousal rating. Affective neurons distribution: valence-related neurons more posteriorly, arousal-related neurons more anteriorly.
*ERP*				
(Buot et al. 2013)^30^	16 PD	STN, ON and OFF med	IAPS	OFF med: ERP larger for unpleasant than neutral pictures. ON med: ERP larger in emotional than in neutral trials in both unpleasant and pleasant tasks.
(Péron et al. 2017)^35^	13 PD	STN, ON med	Emotional acoustic stimuli	Larger ERP happy or angry auditory stimuli compared to neutral stimuli in the right STN.
*LFP*				
(A. A. Kühn et al. 2005)^33^	10 PD (2 with depression, 1 with visual hallucination)	STN, ON med	IAPS	Decrease of alpha for pleasant stimuli condition. Larger desynchronization in trials of pleasant and unpleasant stimuli compared to neutral.
(Brücke et al. 2007)^32^	9 PD (3 had hypomania symptoms after DBS)	STN, ON med	IAPS	Decrease in alpha for pleasant low and high arousal stimuli. Negative correlation between the degree of alpha desyncronization and the valence rating after correcting for arousal.
(Eitan et al. 2013)^34^	17 PD	STN, OFF med	MOntreal affective voice database	Decrease in alpha and low beta in the ventral medial region. Increased spiking activity in ventral medial region. Right > Left STN.
(Chen et al. 2019)^117^	12 patients: 7 PD, 5 ET. 6 patients performed the task before and 6 patients after DBS	PD: STN or GPi; ET: VIM. ECoG strip was targeted to: dlPFC or OFC, or IFC. OFF med	Tap That Emotion, emotional go/no-go task	PD: task related reduced prefrontal theta-alpha and increased prefrontal gamma activity. The high gamma was not restricted to individual contacts of the strip and occurred over a broad cortical region.
(Buot et al. 2020)^31^	15 PD, 7 OCD	STN, OFF med, 7 PD also ON med	IAPS	During image presentation, theta power increased for unpleasant compared to neutral images in both OCD and PD patients.
(Rappel et al. 2019)^25^	307 PD	STN, OFF med	Resting state	High theta-alpha oscillations in the STN ventromedial border which was negatively correlated with depressive symptoms.

*BDI* Beck’s Depression Inventory, *dlPFC* dorsolateral prefrontal cortex, *ERD* event-related desynchronization, *ERP* event related potentials, *GPi* globus pallidus, *IAPS* The International Affective Picture System, *IFC* inferior frontal cortex, *LFP* Local Field Potential, *PD* Parkinson’s disease, *STN* subthalamic nucleus, *OFC* orbitofrontal cortex, *VIM* ventral intermediate nucleus.

#### Emotional processing—within-subject studies

Seven other studies have investigated changes in intracranial recordings from the STN while performing tasks assessing emotional processing^29–35^. These studies also support a modulation of alpha in response to emotional visual stimuli, reporting a greater alpha-ERD and larger evoked response potential (ERP) amplitude in response to pleasant and unpleasant stimuli compared to neutral ones in PD patients ON medication^29–33^. This work has shown that the relationship between alpha and valence holds when controlling for arousal^32^, with valence and arousal likely encoded by separate neuronal populations in the STN^29^. Two further studies specifically investigated hemispheric laterality during emotional acoustic stimuli and demonstrated a regional specialization (ventro-medial) and a hemispheric asymmetry (right dominance)^34,35^, which generalizes to emotional lateralization research outside of PD^36^.

**Fig. 2:**
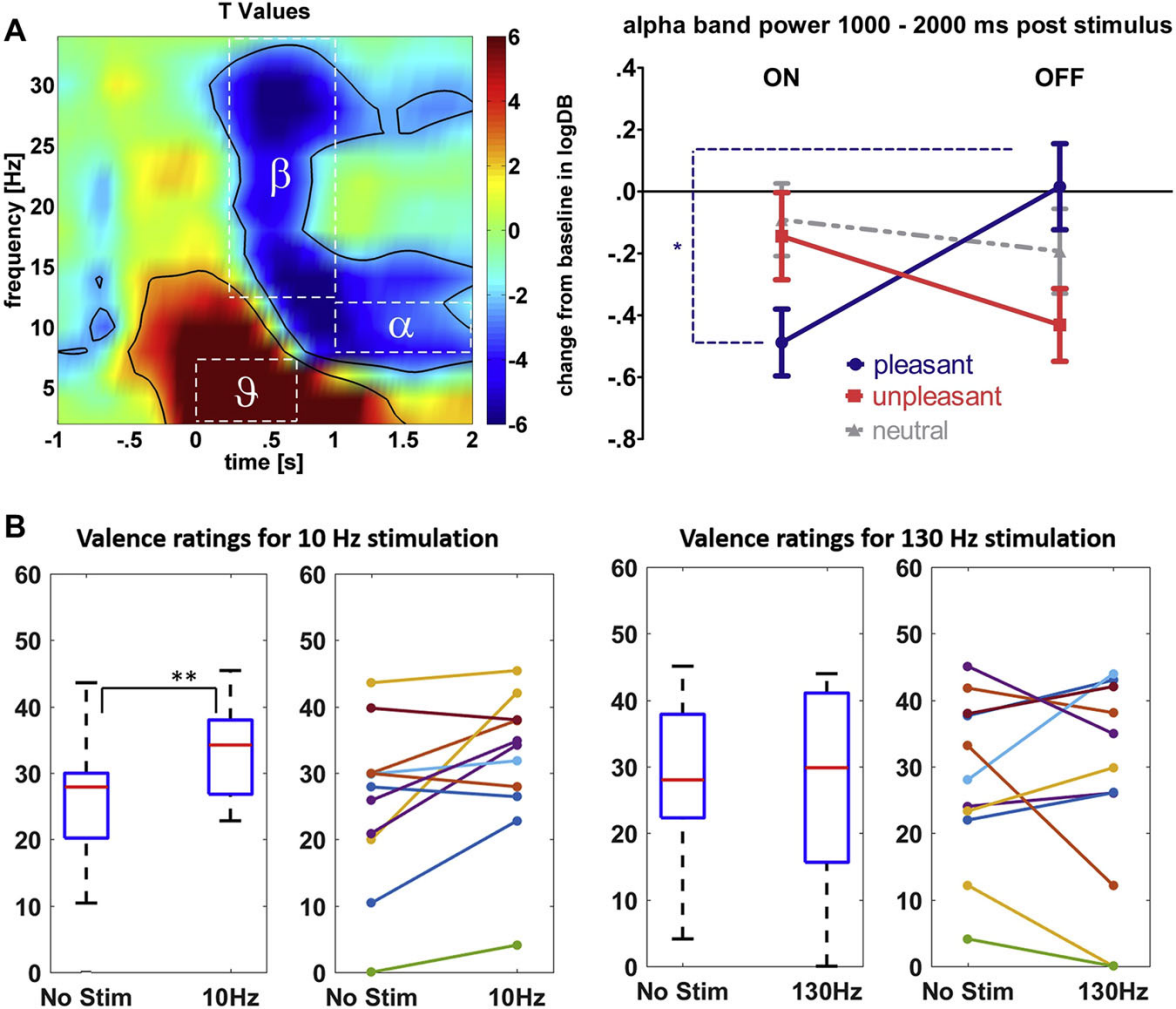
Neurophysiology of affect and emotional processing. **A** Average event locked spectrogram from 28 patients with PD, recorded from the STN at the time of emotional image (IAPS) presentation (left). Statistical parametric mapping on stimulus locked baseline corrected spectrograms demonstrates two significant clusters; a theta power increase and an alpha/beta de-synchronization (left). Alpha is lower (greater desynchronization) following pleasant vs. unpleasant stimuli in the ON medication state (right). This effect reverses OFF medication, showing that in the absence of dopamine alpha is poorly responsive to pleasant stimuli and shows increased reactivity to negative stimuli. (reproduced with permission from Huebl et al. 2014). **B** Impact of alpha (10 Hz) stimulation of the STN increases IAPS valence ratings, an effect not seen with 130 Hz stimulation (reproduced with the permission from Mandali et al. 2021).

Recent investigations have also evaluated emotional processing during stimulation of the STN at different frequencies and provide support that these alpha oscillations may be potentially mechanistic and causal (Fig. 2)^37^. Stimulating the right-STN demonstrated an interaction between emotional picture ratings and stimulation frequency, with 10 Hz (alpha frequency) stimulation increasing subjective valence ratings of negative pictures compared to no stimulation, an effect that was increased in depressed patients^37^. The study however did not measure underlying physiology during stimulation in order to demonstrate entrainment of underlying oscillations to stimulation and therefore only provides partial supportive evidence of causality.

In summary, LFP studies recording from the STN in PD support that a component of alpha band activity in the STN is correlated with processing of emotional stimuli and degree of depressive symptomatology.

#### Behaviour—impulsivity and apathy

ICBs comprise impulse control disorders such as pathological gambling, compulsive shopping, compulsive sexual behaviour and eating, and related disorders such as punding/hobbyism and dopamine dysregulation syndrome^38^. ICB are formally defined as a class of psychiatric disorders characterized by a failure to resist a temptation, urge, or impulse that may harm oneself or others^39^. An interplay between exposure to dopaminergic medications, deep brain stimulation and personality traits, as well as disease-related characteristics in people with PD, appears to underlie the generation of these behavioural disorders^40,41^. Many studies have solely assessed motor impulsivity (using motor stopping tasks) and have reported contradictory results with some studies even finding lower motor impulsivity in certain ICB cohorts^42–44^. It is also worth noting that the compulsivity component of ICB in PD has less commonly been taken into account^45^. Although impulsivity and ICB as clinical constructs encompass many different cognitive subprocesses of decision making, they are likely concentrated specifically in aberrant executive control, reward evaluation and motor control^46^ (Fig. 1, Tables 2 and 3).

**Table 2:** Behaviour—impulsivity and apathy.

Impulsive compulsive behaviour disorders				
Reference	Cohort	Target and medication	Task	Key findings
*Single units*				
(Rossi et al. 2017)^47^	43 PD (12 ICB, 31 no ICB)	STN and GPi, OFF med	Action choice motivated by a monetary reward/loss	PD with ICD: higher STN neurons responsive to reward and less STN neurons responsive to loss, compared with PD without ICD. No difference in GPi neurons.
(Micheli et al. 2021)^48^	24 PD (12 ICB, 12 no ICB)	STN, OFF med	Resting State	ICB+ and ICB− neurons differ in: the fraction of tonic neurons, intra burst frequency, and background unit activity, beta and gamma power. These features correlated with BIS.
*ERP*				
(Eisinger et al. 2021)^53^	39 PD	unilateral STN (18 nuclei) or GPi (28 nuclei), OFF med	Rewarding Go-no Go	GPi: impulsivity (QUIP-RS) was associated with larger responses to reward. STN: impulsivity was associated with smaller responses to reward.
*LFP*				
(Rodriguez-Oroz et al. 2011)^49^	28 PD (ICB PD vs dyskinesia PD vs noICB/noDyskinesia PD)	STN, OFF and ON med	Resting state	Increased theta-alpha in ventral STN coherent with EEG recording in frontal areas in PD ICB.
(Rosa et al. 2013)^51^	17 PD (8 pathological gamblers)	STN, ON med	economic decision-making task with high and low risk options	Increase theta-alpha during task conditions. In PD using a risky strategy (all had PG) the % change in theta alpha power was higher in conflictual stimuli evaluation than non-conflictual ones.
(Mazzoni et al. 2018)^52^	12 PD (6 with pathological gambling)	STN, ON med	economic decision-making task with high and low risk options	Significant difference between conflictual trials ending with a high-risk and those ending with a low-risk decision in the low frequencies LFP, only in patients with pathological gambling.
(Ricciardi et al. 2021)^50^	23 PD (6 with ICB and 17 without)	STN, OFF med	Resting state	A significant positive correlation between 8–13 Hz power and BIS-11score. The correlation was irrespective of the presence or absence of active ICB.
***Impulsivity and apathy—dimensional approach***				
***Executive control***				
*Single units*				
(Zaghloul et al. 2012)^75^	14 PD	STN, OFF med	Probability learning task(training) + decision task (test)	As participants initiated the decision process, there was a consistent and significant increase in spike activity compared to baseline. The level of spiking activity increases with the degree of decision conflict.
(Alanazi et al. 2021)^76^	16 PD	GPi	Oddball task	In response to the deviant tones, the majority (*n* = 51, 82%) of cells showed either an increase or decrease in firing rate; desynchronization in *beta* activity; larger ERP than standard tone.
*LFP*				
(Cavanagh et al. 2011)^67^	8 PD	Study I: ON and OFF DBS while recording EEG. Study II: intracranial EEG from STN, OFF med	Conflict decision making task	Increase in theta in mPFC (EEG) and STN (intracranial recordings) during high-conflict decisions task. Beta suppression and theta enhancement were observed in the STN, during high conflict trials diminishment of low-frequency power across leads.
(Fumagalli et al. 2011)^74^	16 PD (6 had ICB)	STN, ON med	Moral decision-making task	Increased low-frequency oscillation *(5-13* *Hz)* during decision making, more so during the moral conflictual condition than the moral nonconflictual one. Power patterns were not influenced by the presence of behavioral disorders.
(Brittain et al. 2012)^60^	12 PD (11 bilateral STN, 1 left STN)	STN, ON med	Stroop	Theta increases in all task conditions more so in the incongruent.
(Aulická et al. 2014)^63^	4 Epilepsy patients (ACC electrodes) and 3 PD (STN)	STN, ON med	Flanker test	In ACC: Beta: decrease more pronounced in the incongruent trials than in congruent ones. STN: beta and alpha decrease more in the incongruent trials than the congruent ones.
(Zavala et al. 2013)^64^	13 PD (1 with ICD)	STN, ON med	Flanker test	Both congruent and incongruent trials showed an increase in theta; this was higher in the slow-incongruent trials than the congruent ones.
(Zavala et al. 2014)^58^	13 PD (2 with ICD)	STN, ON med	RDK	Increased theta-delta power in high-conflict trials and higher coherence in the theta-delta band between STN and frontal EEG. Activity in the midline frontal cortex was Granger causal to that in STN.
(Herz et al. 2016)^69^	11 PD	STN, ON med	RDK	STN theta and corresponding mPFC-STN coupling are involved in determining how much evidence subjectsaccumulate before making a decision (decision thresholds)
(Zavala et al. 2016)^59^	16 PD	STN, ON med	RDK	Trials with higher levels of ambiguity were associated with increased theta band synchrony between mPFC and the STN, with the cortical oscillations Granger-causal to those of the STN.
(Zavala, Jang, and Zaghloul 2017)^73^	18 PD	STN, OFF med	Working memory	Anterior, lateral PFC and STN showed reduced beta across all trials (both target and distractor), during the distractor ones there was an earlier termination of beta decrease. Elevated beta in STN and PFC during distractor trials were accompanied by elevated level of beta coherence. Significant decrease in spiking activity during each trial compared to baseline.
(Herz et al. 2017)^70^	11 PD	STN, ON med	RDK	2 distinct correlates of decision-making: STN low-frequency oscillatory activity (2–8 Hz), coupled to activity at prefrontal electrode Fz and STN beta activity (13–30 Hz) coupled to electrodes C3/C4 close to motor cortex. The Fz-STN predicted increased thresholds only after accuracy instructions, while the C3/C4-STN predicted decreased thresholds irrespective of instructions, more so during speed emphasis.
(Zavala et al. 2018)^71^	22 PD	STN and subdural strip in mPFC, OFF med	Conflict decision making task	Theta oscillations are coordinated between mPFC and STN when detecting conflict within trial. Across trials, beta increase in PFC were suppressed when the completed trial contained either conflict or error. STN beta power was only modified during the subsequent trial that followed a conflict or error trial.
(Ghahremani, Aron, et al. 2018)^61^	13 PD	STN, ON med	Stroop	Event-related DBS in 4 conditions: no-stim, Ready, Early, Late. Increased low frequencies in the time of conflict detection. Stimulation in this time window reduces RT and increases errors.
(Hell et al. 2018)^72^	6 PD	STN, Embedded device (post operative), OFF med	Modified flanker test	STN low frequencies activity was coherent with frontal midline electrodes (Fz/FCz), alpha/beta oscillations were coherent with those in motor cortical structures. Both cortical and subcortical LFPs correlated with RTs.
(Herz et al. 2018)^72^	7 PD	STN, off stim, continous DBS and Closed-loop DBS	RDK	DBS reduced RT difference between low- and high-coherence trials. Stimulation 400–500 ms after onset of the moving dots cue diminished slowing of RT that occurred on difficult trials off DBS. It also eliminated a relative, time-specific increase in STN beta oscillations and compromised its functional relationship with trial-by-trial adjustments in decision thresholds.
(Wessel, Waller, and Greenlee 2019)^65^	9 PD	STN and M1, ON med	Auditory Simon task	Increase in M1 - STN beta phase locking on incongruent trials. M1 - granger causal to STN.
(Duprez et al. 2019)^62^	16 PD	STN, ON med	Incentive modified Simon task	Motivated cue: increased theta, no effect of size of reward. Decrease in beta, strongest for highest reward. Imperative stimulus: increase in theta, higher for incongruent conditions, no effect of reward. Increase in delta, no effect of congruency or reward. Decrease in beta, stronger for incongruent condition, no effect of reward.

*ACC* anterior cingulate cortex, *BIS-11* Barratt impulsivity scale, *ERP* event related potentials, *GPi* globus pallidus, *ICB* impulsive compulsive behaviours, *ICD* impulse control disorders, *LFP* Local Field Potential, *PD* Parkinson’s disease, *PFC* pre-frontal cortex, *QUIP-RS* Questionnaire for Impulsive-Compulsive Disorders in Parkinson’s Disease–Rating Scale, *RDK* Random Dot Kinematogram, *STN* subthalamic nucleus.

**Table 3:** Valuation, motor control and learning.

Reference	Cohort	Target and medication	Task	Key findings
***Subjective valuation***				
*Single units*				
(Howell et al. 2016)^85^	4 PD, 1 MSA, 3 dystonia	GPi, OFF med	Monetary Incentive Delay task	Limited responses (2/35, 5% of neurons) only to null and lose signals, no to reward. 1 neuron increased firing rate for loss compared to win; 1 neuron responded to null cues while being inhibited by loss.
(Justin Rossi et al. 2017)^83^	50 PD (20 with unilateral STN and 30 with unilateral Gpi)	STN and GPi, OFF med	Reward task	In STN: more neurons responded to reward than to loss. STN neurons were valence specific (they responded either to reward or loss). In GPi: the proportion of neurons responding to reward was similar to that responding to loss
(Al-Ozzi et al. 2021)^86^	12 PD	STN, OFF med	Decision-making using a 2-choice simple preference-based task.	ERD in beta activity, and change in spiking activity of single cells (increase or decrease) in response to favorite picture compared to non-favorite ones
*LFP*				
(Zénon et al. 2016)^80^	14 PD	STN, ON and OFF med	Effort and reward based decision making task	Response to both reward and effort was in the 1–10 Hz range; larger response for larger reward. Larger responses led to higher chances of accepting the trial (no effect of medication status). LFP amplitude decreased for larger effort intensities; LFP changes to effort predicted the acceptance rate (stronger in the ON DOPA condition). Low-frequency LFP correlated with the magnitude of pupil response.
(Pearson et al. 2017)^81^	15 PD	STN, OFF med	BART	Single unit recording: local firing rates are sensitive to reward and risk. LFP: For successful stops, theta increases preceding stop movements, this is not the case for unexpected stop (popped baloon)
***Motor control***				
*ERP*				
(Sauleau et al. 2009)^118^	14 PD	STN, ON and OFF med	‘Standard motor’ task, a ‘control motor’ task, a ‘counting’ task	3 epochs over the 760 ms following the onset of the warning cue were identified: the second and third epochs of evoked LFP were significantly influenced by behavioural relevance (type of cue) and medication state (increased ON levodopa)
(Chen et al. 2020)^100^	21 PD	STN, GPi and VIM + ECoG strip in IFC, OFF med	SST	Cortical ERP by STN stimulation revealed short latency events. Stopping-related potentials in the cortex preceded stopping-related activity in the STN, and synchronization between these task-evoked potentials predicted the stop signal RT.
*LFP*				
(Williams et al. 2003)^92^	9 PD	STN, OFF and ON med	SST	LFP activity in the beta frequency band (~20 Hz) was modulated by the behavioural relevance of theexternal cue.
(Andrea A. Kühn et al. 2004)^91^	8 PD	STN, OFF and ON med	Go No-Go	Go trials: beta decreased prior to movement with onset latency correlated with RT, then beta increased again. No go trials: beta decrease following imperative signals was prematurely terminated compared with go trials and reversed into an early beta power increase.
(Ray et al. 2012)^93^	9 PD	STN, ON med	SST	Go-signals were followed by a reduction in beta and an increase in gamma. Stop-signal, unsuccessfully stopped: initial beta increase followed by subsequent desyncronization; successfully stopped: same as unsuccessfully but less beta desyncronisation; gamma increase particularly when inhibition is failed.
(Alegre et al. 2013)^94^	10 PD (4 had ICB at the moment of surgery)	STN, OFF and ON med	SST	Decreased beta after the go signal in al conditions. Go and failed stopped had a subsequent beta rebound, more marked in the OFF med. Successfully stopped had a subsequent return of beta to baseline. Increased gamma in the ON med in go and failed stopped conditions; decreased gamma in the successfully stopped. Increased theta in the 3 conditions both ON and OFF. Gamma decrease during the successfully stopped condition was absent in 4 patients with ICB.
(Benis et al. 2014)^95^	12 PD	STN, ON med	SST	*Reactive inhibition*: decreased beta in GO trials and successful STOP. The decrease was lower in Successful STOP trials than GO trials. The decrease of beta was of lower and was interrupted earlier in successful than unsuccessful stopping. *Proactive inhibition*: decrease in beta in all trials, but beta decrease was greater during GoFast trials than during GO trials.
(Wessel et al. 2016)^97^	9 PD	STN, ON med	SST vocal, TMS	Beta increased and MEP amplitudes reduced during successful stopping. Negative correlation between single-trial STN beta power and MEP amplitude.
(Fischer et al. 2017)^99^	9 PD	STN, ON med	Finger tapping with Stop signal	STN gamma (60–90 Hz) increased most strongly when the tap was successfully stopped, whereas phase-based connectivity between the contralateral STN and motor cortex decreased.
(Ghahremani, Wessel, et al. 2018)^97^	16 PD (8 for the manual SSRT and 8 for the vocal SSRT), 1 dystonia. Part of the sample is included in Wessel et al.	STN, ON med	SST manual and vocal	Beta power was greater in successful stop than failed stop for both manual and vocal tasks. For the manual task, beta decreased in the failed stop trials and increased in successful stop trials. For the vocal task: low beta was higher during successful stop than failed stop in the right STN only. Alpha peaked after Go cues, but remained elevated only on slow go trials in both vocal and manual tasks.
(Wessel, Waller, and Greenlee 2019)^65^	9 PD	STN and M1, ON med	SST	Increase in M1 - STN *beta* phase locking on incongruent trials. M1 - granger causal to STN.
***Feedback, learning and updating***				
(Schroll et al. 2018)^105^	19 PD	STN, ON med	Reinforcement learning	Beta range oscillations respond to reward but not to reward prediction error. Alpha and lower frequencies respond to previous reward from trial before.
(Skvortsova et al. 2021)^106^	3 PD	Bilateral PPN, ON med	Instrumental learning task	Behavioural data showed that the cue with the higher probability of reward (75%), was more frequently selected on average. LFP: in response to reward outcome (but not to no-reward outcome) there was an increased power in low-frequency bands (10-20 Hz alpha-beta)

*BART* bart balloon analogue risk task, *ERP* event related potentials, *ERD* event-related desynchronization, *GPi* globus pallidus, *IFC* inferior frontal cortex, *LFP* Local Field Potential, *M1* motor cortex, *MEP* motor evoked potential, *MSA* multiple system atrophy, *PD* Parkinson’s disease, *PPN* pedunculopontine nucleus, *RT* response time, *SST* Stop Signal Task, *STN* subthalamic nucleus, *TMS* transcranial magnetic stimulation, *VIM* ventral intermediate nucleus.

#### Impulsivity and apathy—across subjects studies

Single unit recordings in people with PD have implicated the STN in patients with ICB^47^, however, the spectral components of micro-electrode single unit activity have been less consistently linked to ICBs in a single frequency band^48^. The first study evaluating neural mechanisms of ICB in PD using LFP recordings identified low frequency (4–10 Hz) oscillations as being linked to ICBs, particularly in the ON medication state and in ventral STN^49^. This study also found greater STN-premotor/prefrontal (EEG contacts F3,F4) cortex theta (4–7 hz) band coherence in patients with ICBs compared to those without. However, other frequencies have also been implicated and recently a correlation has been shown between resting state low frequency activity in the alpha range during the OFF medication condition and trait impulsivity as measured with the Barratt Impulsivity scale irrespective of the presence and severity of ICB^50^.

Two studies have investigated differences in STN LFPs in PD patients specifically with pathological gambling, using behavioural paradigms involving economic decision-making^51,52^. Spectral analysis revealed a relationship between pre-cue beta and diagnosis of ICB, but this difference did not predict future choices^52^. However, all PD patients showed an STN low frequency (2–12 Hz) increase during decision making and PD patients with pathological gambling adopted a risky strategy and showed greater, low-frequency power during high conflict trials^51^. Clinical impulsivity also predicts subcortical evoked response activity and shows dissociable effects on STN and GPi^53^.

In summary, these studies demonstrate that STN and GPi recordings can elicit ICB biomarkers at the levels of single cells, ERPs and LFP oscillations. There is a suggestion that low frequency local activity, particularly a component in the theta range, may be particularly associated with ICBs in PD.

#### Impulsivity and apathy—within-subject studies

##### Executive control and reflection impulsivity

Appropriate executive control prevents reacting to inappropriate cues, responding when too little information is available or waiting too long to respond. Deficits of this type of information processing can result in reflection impulsivity^40,54^. There are a number of behavioural paradigms which evaluate action inhibition under conflict which can index higher order executive control, including the Stroop task^55^, the Flanker task^56^, the Simon task^57^ and random dot kinematograms (RDK)^58,59^.

LFP studies in PD using the Stroop task, in which words are presented in congruent and incongruent colours, demonstrate a relative increase in theta activity on incongruent trials^60,61^. Event-related STN stimulation during the Stroop task has probed the temporal window of conflict processing and demonstrated that stimulation delivered early post stimulus causally impacts behaviour, relatively speeding up responding on conflict trials and increasing errors^61^. Generalizing to alternative forms of inhibitory control, other studies have recorded STN LFPs during spatial conflict tasks (Simon task and Flanker tasks) to demonstrate increased theta power during spatially incongruent trials, with rewarded inhibition linked to theta phase resetting (indexed by inter-trial theta phase clustering)^62–65^. Cross-site phase locking between motor cortex (M1) and STN has also been shown to regulate the influence of the STN on M1 representations of incorrect response-tendencies, in the beta band during the Simon task^65^. These results demonstrated that theta activity is linked to conflict processing, through both theta amplitude and phase modulations, likely supported by signal processing in other frequency bands, including the motor cortex - subthalamic beta network.

**Fig. 3:**
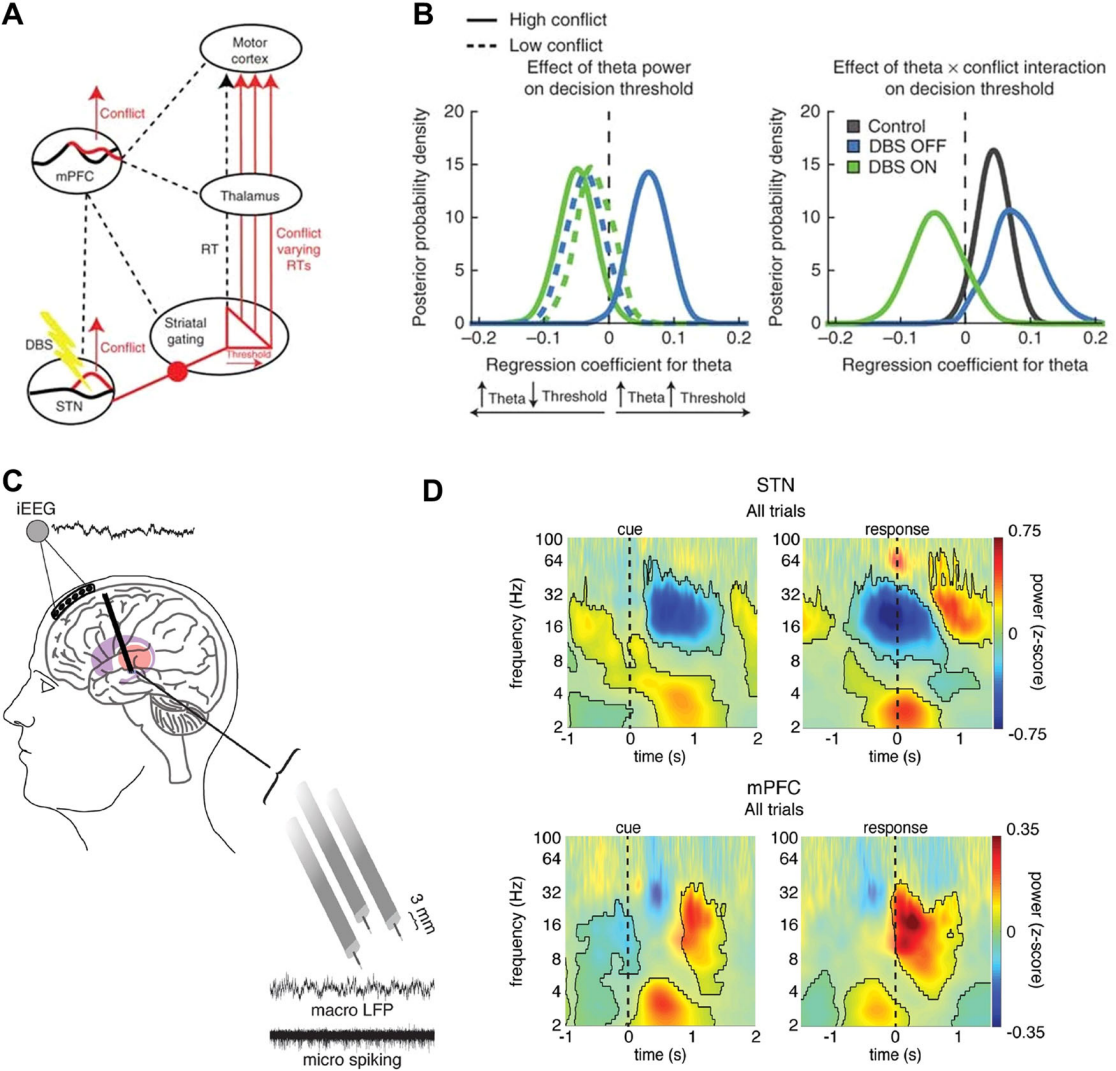
Neurophysiology of executive control and conflict. **A** Theoretical model of medial prefrontal cortex (mPFC)—STN mediation of decision thresholds. mPFC detects conflict which leads to adjustment of decision thresholds in the STN. This process is interrupted by DBS. **B** Modelling of relationship between mPFC theta and decision threshold (negative regression coefficient—high theta correlates with low decision threshold, positive regression coefficient - high theta correlates with increased decision threshold). This reveals that OFF DBS (blue line)—increased theta was associated with increased decision threshold for high-conflict trials (solid line), but not low-conflict trials (dashed line). ON DBS, increased theta was associated with a decreased decision threshold on high-conflict trials (reproduced with permission from Cavanagh et al. 2011). **C** Schematic of combined intracranial mPFC—STN intra-operative electrode recordings. **D** Normalized oscillatory power averaged across all STN (upper) and mPFC (lower) electrodes and across all correct trials in a movement conflict task. Both brain regions showed a pre-response increase in theta power, although this is earlier and greatest averaged to the cue in the case of mPFC. In the beta band, the STN showed a pre-response decrease in power while the mPFC showed a post-response increase in beta power (reproduced with permission from Zavala et al. 2018).

Recent work has combined the Flanker task with drift diffusion modelling (DDM), a computational framework that models decision making as a process of evidence accumulation towards a threshold. This found that some STN theta activity is linked to dynamic decision processes, rather than being simply a biomarker of conflict, with subthalamic stimulation modulating evidence accumulation rates and theta setting thresholds^66^. A further seminal study extends this work to other forms of conflict by using a paradigm that presents decisions containing either high or low reward conflict while recording from medial prefrontal cortex (mPFC) and STN (Fig. 3)^67^. This study again confirmed an increase in mPFC and STN theta activity which, after fitting the DDM, was found to be correlated with decision thresholds.

One challenge for behavioral tasks that present abrupt cues is the profound evoked low frequency neural activity that any salient sudden cue can elicit, potentially confounding salience with conflict. A continuous form of conflict paradigm implements an RDK in which clouds of dots continuously move in different directions on the screen to, on average, indicate the correct direction for an upcoming voluntary movement such as a button press^68^. In addition to investigating conflict (with dots moving in different directions), the paradigm measures reflection impulsivity since subjects control how much information they sample before making a decision. RDK paradigms plus neurophysiology recordings in PD have confirmed increased low frequency activity during motion conflict, activity that was coherent between medial prefrontal cortical (mPFC) theta and STN theta-delta in conflict trials^58^. Prefrontal cortical - STN theta dynamically tracks evidence accumulation, accounting for variable incoming evidence presentation rates^59^. Combining RDK paradigms with DDM has again demonstrated that trial-by-trial decision thresholds are also indexed by mPFC-STN low frequency phase alignment^69^. Using RDK paradigms it has also been shown that mPFC-STN theta appears to again be supported by a spatially and spectrally segregated beta band neural network between motor cortex and STN which also supports decision threshold setting^70^. This study showed that these two networks were functionally distinct in mediating speed - accuracy tradeoffs. Specifically, an increase in STN low frequency oscillatory power was found to predict increased thresholds only after instructions emphasizing accuracy, while cue-induced reductions of STN beta reflected a decreased threshold, irrespective of whether the subject was prioritising speed or accuracy.

In addition, recent work investigating mPFC-STN theta and beta within a novel conflict paradigm showed that functional roles of these oscillations are regionally specific (Fig. 3)^71^. The authors here demonstrated that within trial adaptations to conflict are mediated by increased mPFC-STN theta synchrony, causing response inhibition, whereas across trials, beta signals more strongly mediated behavioural adaptations to conflict or errors. Here, beta was modulated within a trial in the STN, whereas more so in PFC post trial. Support for a causal role for a component of beta oscillations in threshold setting comes from a study which combined RDK, DDM and closed-loop DBS in a single paradigm to demonstrate a temporally precise causal impact of DBS and beta oscillations on response threshold setting^72^. Notably, the component of STN beta activity coupled with the prefrontal cortex (PFC) may have a complementary, more general role to motor initiation threshold setting, as parallel work has demonstrated that this network indexes working memory encoding, even in the absence of motor initiation^73^. Theta signals have also been linked to higher order conflict resolution including moral reasoning^74^. These data are anatomically complimented by studies of single units recording, where a change in neural firing activities was described in the STN and in the GPi in response to conflict in a decision-making task^75^ or to a deviant tone in an oddball task^76^.

In summary, these studies demonstrate a link between a component of PFC theta activity and PFC-STN synchrony in conflict detection, in addition to complementary effects of a beta motor cortex - STN network. Further work investigating how these two networks collectively set thresholds for action initiation are warranted.

##### Subjective valuation: reward cost evaluation and choice impulsivity

One of the most studied cognitive functions of the basal ganglia in animal models is reward processing^77^. In humans, reward processing and apathy have been mapped to distributed prefrontal cortical and basal ganglia networks with ventromedial prefrontal, anterior cingulate cortex, supplementary motor areas and ventral striatum plus connected areas being important nodes^5^. Electrophysiological studies in PD have further expanded our knowledge on the role of basal ganglia in reward processing in humans using paradigms for reward, loss and effort evaluation during recordings from STN, GPi and PPN (Table 3).

**Fig. 4:**
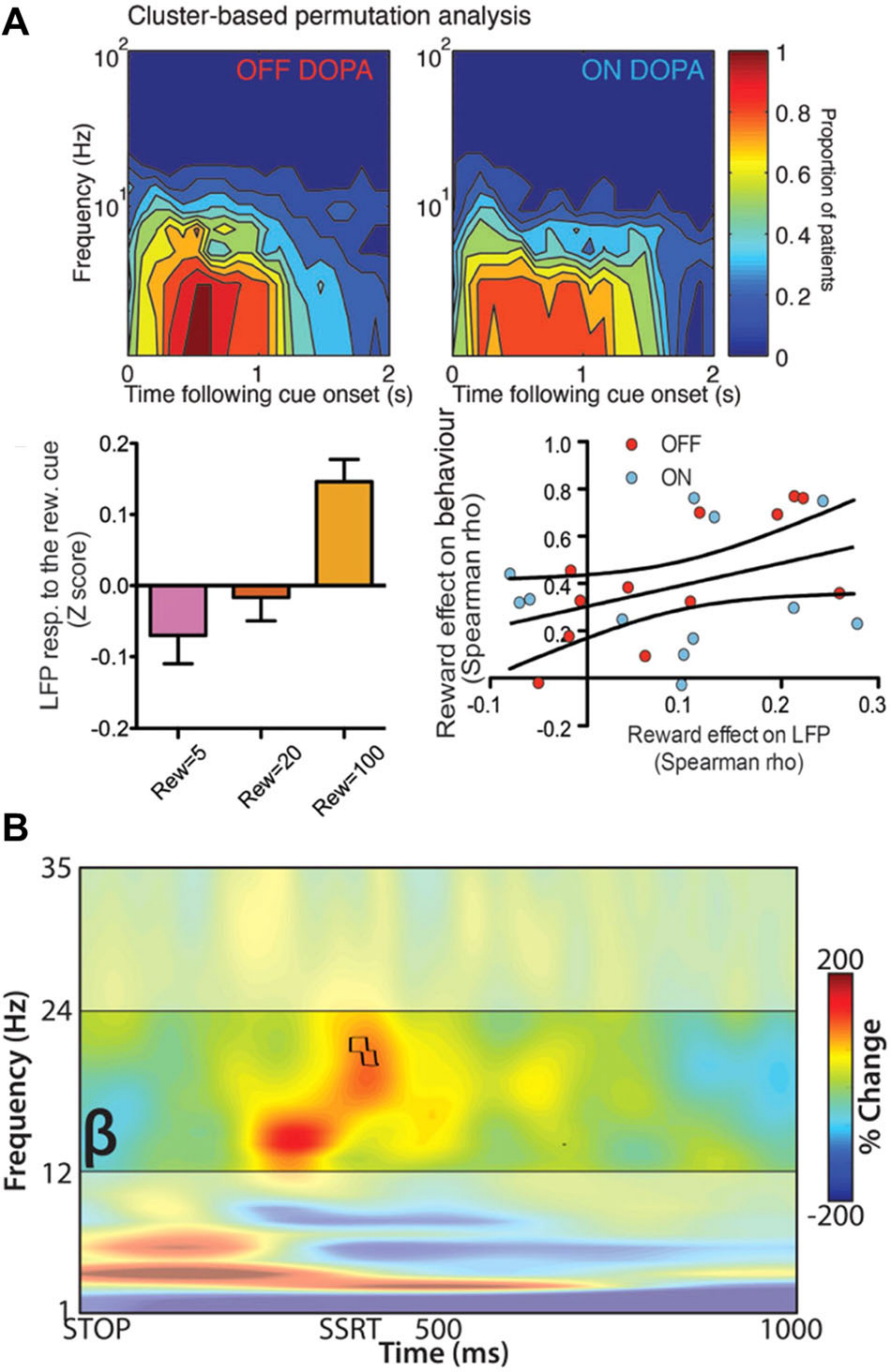
Neurophysiology of reward and action inhibition. **A** STN group level neurophysiology related to reward-effort decision making. Cluster-based permutation analysis showing proportion of subjects in which the LFP power increased significantly for each frequency x time combination, demonstrates low frequency reward locked STN activities (top). Low frequency activity scales with reward value and notably the reward effect on LFP correlates with the reward effect on behaviour (reproduced with permission from Zenon et al. 2016). **B** Action stopping indexed through a stop-signal task during LFP recordings from the STN. Stopping triggers an increase in STN beta activity which starts prior to the stop signal reaction time (reproduced with permission from Wessel et al. 2016).

Reward versus effort trade-off paradigms, where participants make decisions about whether to exert different levels of effort for reward, are valuable to understanding healthy motivation and the clinical symptom of apathy, which likely reflects modulation of sensitivity to effort and reward^78,79^. The first comprehensive study in PD, using a reward (monetary) and effort (grip force) paradigm^80^, reported an STN spectral response to both reward and effort cues in the 1–10 Hz range with larger responses produced by larger rewards. Moreover, these responses were reflective of the subjective value of reward and predicted patients’ trial-by-trial decisions of whether to exert effort for reward, an effect that weakened OFF levodopa (Fig. 4)^80^. Low frequency activity also appears linked to other types of reward discounting, as another study analysing LFPs recorded from the STN showed an increase in theta power preceding risk taking decisions^81^. Further studies of single unit activity and LFP recorded in the STN of patients with PD have used reward paradigms including the Balloon Analogue Risk Task^82^ (for risk taking behaviour), a rewarded Go/NoGo task^83^, a modified version of the Monetary Incentive Delay task^84,85^(reward evaluation) and a task of decision making using a 2-choice preference-based task^86^. These studies suggested that STN cells show significant modulation by reward and that STN neurons that responded were valence specific (i.e., they responded exclusively to either reward opportunity or threat of loss), moreover they suggest that the STN is modulated more in response to reward than the GPi.

In summary, single unit recordings and LFP studies in the STN and GPi in PD support that particularly the STN is involved in the processes of reward, risk, and effort evaluation and that this may involve a component of low frequency (particularly theta) activity.

##### Motor control and motor impulsivity

Information processing and reward evaluation lead to motor preparation and initiation. The computational process of action control involves the appropriate release of prepared movements paired with stopping or switching when required. Behavioural tasks can be used to assess impulsivity defined as impaired inhibitory control over prepotent motor or cognitive responses. Processes of action selection versus action inhibition have been shown to map to dissociable subcortical locations of dorsal and ventral STN respectively^87^. The GPi, although less studied, also appears to encode motor versus higher order functions topographically^88^.

In the motor Go/No-Go task^89^ and the Stop Signal Task (SST)^90^, participants are presented with a stimulus that requires them either to respond (Go) or withhold a response (NoGo/Stop). Impulsive participants may make more commission errors (Go responses on No-go or Stop trials) and/or anticipation errors (responding too fast). A number of LFP studies have probed the role of STN in modulating action inhibition using these tasks with the majority taking a within-subject approach. It is now increasingly recognized that motor impulsivity is just a single component of impulsive behavior and may not correlate strongly with clinical impulsivity symptoms^44^.

Three studies have employed simple or modified versions of the Go-NoGo task^83,91,92^ while six studies have employed the SST^65,93–97^ with concurrent STN LFP recordings. In the first classical Go-NoGo study, beta oscillations decreased prior to movement and rebounded following post-movement in the Go trials^91^. However, in the No-Go trials, the beta decrease terminated earlier and before the reaction time in a majority of patients. This was supported in a modified, probabilistic, bimanual version of the Go-NoGo paradigm in which patients had to switch hand movements^92^. A third, recent Go-NoGo study was more complex, in that there was an additional monetary reward/loss component incorporated into the design^83^. This was a single unit recording study in both STN and GPi and mainly focused on reward related neural findings, but did report that reward related firing rates were not differentially modulated by motor initiation or inhibition.

The SST paradigm, in which the stop signal comes after the initial go signal at a variable delay, is supported by a computationally described analysis framework—the race model^98^. The first study to investigate STN LFP signals during the SST showed an increase in beta oscillations after the stop signal which was higher on successful, compared to failed stops^92^. This has been replicated in other studies, related to both reactive and proactive inhibition and in a vocal stopping task which found that stopping related beta changes are right lateralized (Fig. 4)^93,95,96^. A further study which did examine for across-subject symptom differences using classical categorical symptom classification, showed a reduction in beta desynchronisation (a relative increase) in successful stopping^94^. However, this study also showed that in the ON medication state, successful inhibition of the response was associated with a decrease in cortico-subthalamic gamma power and coherence. This gamma related finding was notably only present in the 4 out of 10 subjects with dopamine agonist related ICDs but hasn’t been replicated in other studies which found conflicting results^99^. Recent work has also shown that beta activity is likely complemented by rapid evoked responses through the hyperdirect pathway from prefrontal areas including the right inferior frontal gyrus for mediating response inhibition^100^.

Motor inhibition following prepotent action planning and initiation is a final stage of response control. It is here shown to be partly related to motor network beta signals, with an increase in beta, multiplexed with other signals, being found at the time of motor inhibition and associated with successful stopping.

##### Reward learning and updating

Whilst there have been a number of studies investigating motor learning and outcome related updating^101–104^, to date there have been very few studies investigating reward learning, with many reward studies only reporting on the reward decision making epoch rather than post-feedback physiology^67^. One study directed towards reward feedback and learning investigated whether beta oscillations are specifically modulated by outcomes in a reinforcement learning task^105^. In the task, subjects made self-directed joystick movements that were then mapped onto dynamic reward probabilities that had to be learnt. This study found that although STN alpha and low beta activity was negatively correlated with previous reinforcement magnitudes, these did not specifically correlate with reward prediction errors (RPEs). This is somewhat surprising given the link between beta oscillations and dopamine, which also signal RPEs^77^. This might be partially explained by dissociated effects of phasic and tonic dopamine neuronal firing or to specifics of the task and recording / processing techniques. Notably, one recent study evaluated LFP changes during a reward task, but this was in only three PD patients undergoing DBS of the PPN^106^. They showed that in response to reward outcome (but not to no-reward outcome) there was an increased power in alpha-beta bands (10–20 Hz) in PPN^106^, supporting further reward and RPE related research in PD.

A fuller account of the neurophysiology and neural signals of reward and outcome learning is needed to fully capture the time varying changes in NPS seen in PD over time, particularly the transition from simple impulsive to compulsive behaviours^45^.

## Discussion

Non-motor symptoms and specifically NPS in PD are common and highly disabling, yet, their pathophysiological basis is unclear and the impact on these symptoms of pharmacological and surgical treatments are still actively debated^107,108^.

In this review we examine previous studies employing intracranial neurophysiology in patients with PD to explore biomarkers of mechanisms underlying the generation and maintenance of mood, motivation and behavioral symptoms. We highlight behavioural dimensions that are particularly pertinent to PD with specific reference to 1) emotional processing, 2) executive control 3) subjective valuation (reward and cost evaluation) 4) motor control and 5) learning and updating.

The available data suggest that there are segregated spatio-spectral neural networks within the brain that may partially index separable dimensions of cognitive sub-processes underpinning mood, motivation and behaviour. Emotional information appears to involve a component of the alpha oscillations expressed in the STN. Executive control and more specifically conflict detection shows an association with pre-frontal theta activity synchronized to a component of STN theta activity. There is also evidence that subjective valuation (in terms of reward, loss and effort) is also partially mediated through low frequency oscillations - mainly a theta component. Regarding motor control and action inhibition, the evidence supports a link between motor cortical - subcortical beta and motor inhibition, potentially multiplexed with gamma. The presence of different neural networks, indexed by different spatial and spectral patterns, for the different cognitive dimensions suggests that these mechanisms are likely computed through different networks. However, although the evidence suggests associations between different spatio-spectral networks and underlying cognitive subprocesses, we do not expect there to be perfect or simple one-to-one mapping between a neurophysiological signal and a complex computational process.

The research to date has highlighted potential biomarkers for future neuropsychiatric state tracking in PD that could be applied to personalized, adaptive, DBS therapies. The studies also show how different cognitive components of high dimensional clinical constructs, such as impulsivity or apathy, appear to be subserved by spatially overlapping but spectrally segregated networks. This is well illustrated by the medial prefrontal - subthalamic network in the theta range that subserves components of impulsivity and can be mapped to underlying computational processes including decision threshold setting. This can be contrasted with a motor cortical area - subcortical beta network which relates to action control and inhibition. Therefore, although spatially overlapping, these networks can be segregated through spectral features which can be used to identify cortical to subcortical distributed networks related to specific cognitive processes. We propose this approach could be used to further identify other spatio-spectral networks, related to distinct cognitive subprocesses, including for example reward (prefrontal cortex - basal ganglia) or effort (supplementary area - basal ganglia) networks. Furthermore, and more broadly, this supports a model whereby cortical information from segregated regions, subserving different cognitive functions, are funnelled into the basal ganglia, but remain partially segregated via dissociable temporal dynamics, indexed through spectral properties. Interactions between these spatially overlapping, but spectrally distinct networks, may be served by higher order inter-frequency interactions in basal ganglia cortical connections towards integration and action selection^109^. This spatio-spectral network model can complement and also likely integrate with rate firing circuit models, including for example classical prefrontal-basal ganglia parallel organization models and direct versus indirect within basal ganglia accounts^23,110^.

### Limitations and suggestions for behavioural neurophysiology in PD

Although promising, computational neurophysiology aimed at understanding fundamental mechanisms of motivation and its dysfunction is still at an early stage. A key issue relates to the specificity of identified biomarkers for specific symptoms, NPS and cognitive sub-processes. The data to date supports the hypothesis that there may be meaningful and clinically useful segregation between specific spatio-spectral networks with dissociable cognitive subprocesses. Neurophysiological recordings, with high spatial and temporal resolution, significantly increase the specificity of mapping from neural activity to NPS in PD compared to alternative techniques. However, single site LFP recordings, even in narrow power bands may contain activity from multiplexed underlying neuronal pools linked to different networks. Low frequency activity particularly has more spatial spread which could exacerbate this mixing of signals. Therefore, at a given site, there is not likely a simple one-to-one mapping between an oscillatory frequency band and a symptom or behavioural mechanism. We therefore recommend recording of multi-site (cortical and subcortical) LFPs where possible to investigate spatio-spectral networks rather than simply trying to relate single-site single power bands to individual symptoms of computational processes. Furthermore, many of the underlying dimensions and associated behavioural paradigms are not perfectly orthogonal to each other and therefore will correlate with multiple neural activities.

It is important to stress that future studies should focus on decorrelating the contributions of different dimensions of motivation related cognitive processing (including motor processes such as vigour), given that NPS are highly correlated. This could be achieved by testing multiple different behavioural paradigms together in a single cohort in order to examine for specificity of correlations or through using computational models that can isolate separable latent cognitive variables. Notably a majority of studies have been recorded from the STN, rather than GPi, and yet research to date suggest that these two nodes are functionally dissociable and therefore further interrogation of the GPi (and other targets) will be valuable. Intracranial physiology is also currently restricted to PD patients with relatively long disease duration and severe motor symptoms as inclusion criteria for DBS and caution is warranted before extrapolating these findings to all patients with PD. However, pre-operative neuropsychiatric evaluation of DBS patients selects for patients without extremes of NPS which supports a degree of generalization.

While there is a wealth of behavioural research in PD, there is a relative lack of studies specifically designed to investigate neural biomarkers of NPS based on categorical assessments of clinical severity. Integrating the categorical and dimensional approaches and investigating the role of dopaminergic medications through longitudinal (within-subject) studies will help reveal more about inter-individual and the intra-individual differences. Also, this will clarify whether neural correlates identified are secondary to the symptom themselves (states) or related to predisposing factors that make certain PD patients more prone to develop symptoms (traits). Further, there is a current lack of research investigating the underlying neurocircuitry and neural signals of reward learning in PD, which could be critical to understanding the longitudinal evolution of behavioral symptoms in PD, particularly the progression towards more compulsive, habitual responding^111,112^. A progression from action, to habits and then compulsions in PD shows parallels to processes that have been mapped out in addiction processes^113^.

### Towards future principled therapies for neuropsychiatric symptoms

Previous research findings using intracranial recordings have been used to guide treatment developments for motor symptoms in PD, including the use of STN beta power as a neural marker to drive adaptive DBS stimulation in PD for bradykinesia and rigidity^15,114–116^. Adopting the same approach for non–motor symptoms and identification of the precise neural markers of NPS could be used in the future for closed-loop therapy for mood, motivation and behavioral symptoms in PD. The recent release of new commercial DBS devices that sense neural signals as well as having the capability to deliver precisely temporally targeted stimulation according to neurophysiological biomarkers opens a direct and potentially rapid translational pipeline to leverage this emerging knowledge base^32^.

PD has been described as the quintessential neuropsychiatric disorder^2^. Using neurophysiological approaches combined with dimensional paradigms and categorical classification, we have the potential to uncover fundamental neurophysiological mechanisms of mood, motivation and behavioural symptoms in PD. This understanding could then be used towards the characterization of accurate biomarkers and principled therapy developments in PD, and further applied to a wide range of other neuropsychiatric disorders.

## Data Availability

All the data presented as part of the manuscript are already available and published in the literature. No datasets were generated or analysed during the current study.
